# Stop Fooling Yourself! (Diagnosing and Treating Confirmation Bias)

**DOI:** 10.1523/ENEURO.0415-24.2024

**Published:** 2024-10-16

**Authors:** Richard T. Born

**Affiliations:** Department of Neurobiology, Blavatnik Institute, Harvard Medical School, Boston, Massachusetts 02115

**Keywords:** blinding, cognitive bias, confirmation bias, experimental design, masking, randomization

## Abstract

Confirmation bias (CB) is a cognitive bias that allows us to fool ourselves by selectively filtering data and distorting analyses to support favored beliefs or hypotheses. In this article, I will briefly review some classic experiments from cognitive psychology that illustrate what a powerful, pernicious, and insidious force CB is. I will then discuss how to recognize CB in our own thinking and behavior and describe specific elements of good experimental design that can mitigate its effects. These elements—such as randomization and blinding—are conceptually straightforward but often difficult in practice and therefore not as widely implemented as they should be.

## Significance Statement

I provide a brief tutorial on the nature of confirmation bias and how its detrimental effects can be mitigated by randomization and blinding.

## Our Biased Brains

“The first principle is that you must not fool yourself—and you are the easiest person to fool.”

—Richard Feynman, “Cargo Cult Science”

**Figure 1.**
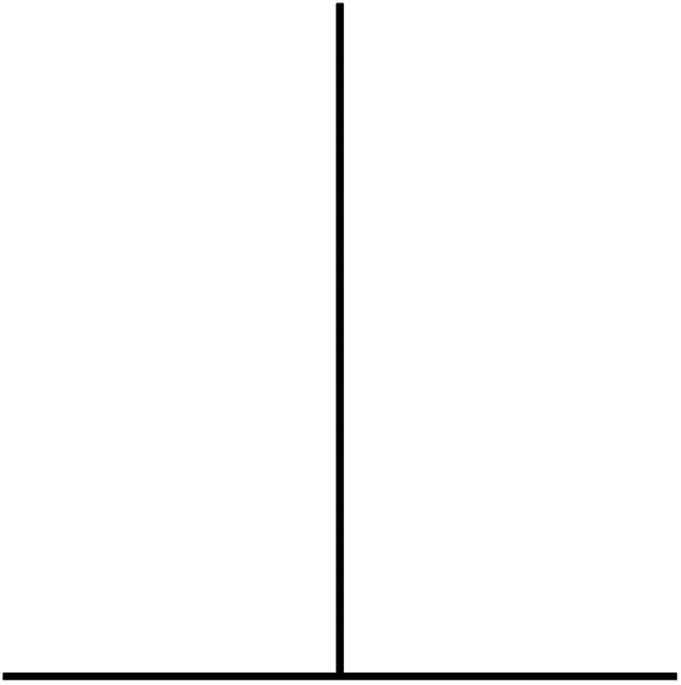
Which line is longer?

Our most important laboratory instrument is our brain. The human mind is a powerful engine for creativity and problem-solving, and we normally think of it as a faithful tool for discovering truths about nature. But it turns out that our brains are not quite as reliable as we believe—this starts with very basic operations like visual perception. For example, in the graphic shown in Figure 1, which line is longer? Virtually all human observers report that the vertical line appears longer, when, in fact, they are exactly the same length (Wolfe et al., 2005). To me, the vertical line appears to be much longer—not even close to the horizontal. After I created the figure, saved it in a suitable format, and pasted it into this document, I felt compelled to recheck the length. I was no longer sure that the aspect ratio hadn't changed and made the horizontal line actually shorter. But no.

**Figure 2.**
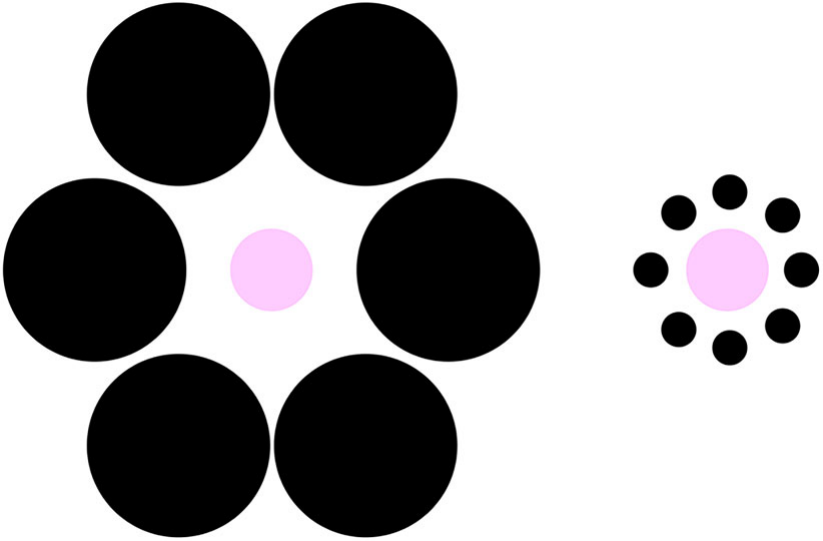
The Ebbinghaus illusion. Which pink circle is bigger?

Such visual illusions aren't rare. Figure 2 shows another famous comparison called the Ebbinghaus illusion: Which of the two pink circles is larger? Most observers report that the left-hand pink circle appears smaller than the one on the right. But as you've probably guessed by now, both pink circles are exactly the same size. Such misperceptions—or, more accurately, perceptual biases—don't just apply to visual attributes like length and size; they can also be demonstrated for brightness, color, motion, and depth (Gregory, 1997). These biases inform us about the kinds of algorithms that our visual systems use (Weiss et al., 2002), and they have been of great use to visual scientists trying to “hack” the visual system.

Fortunately, we have good tools for defeating the kinds of biases depicted in Figures 1 and 2: we have rulers that we can use to make measurements, revealing the quantitative equivalence of the line lengths and the sizes of the pink circles. But sensory biases are only the beginning. Our brains are also subject to higher level, cognitive illusions that distort how we actually think: how we ask questions, how we make observations, how we gather information, and how we analyze data (Kahneman and Tversky, 1996). It is one such cognitive bias, known as confirmation bias (Nickerson, 1998), that this article will explore in some detail. After briefly defining a few terms that are essential for any discussion of bias, I will review some classical experiments that help make us aware of just how pervasive and pernicious confirmation bias can be. Merely being aware of our biases can help us combat them, but I will also describe specific methods, such as “blinding” (or “masking”), that have been developed to mitigate the effects of our intrinsic cognitive biases. Many of the practices we consider good experimental design are useful in preventing, or at least minimizing, the influence of confirmation bias.

**Figure 3.**
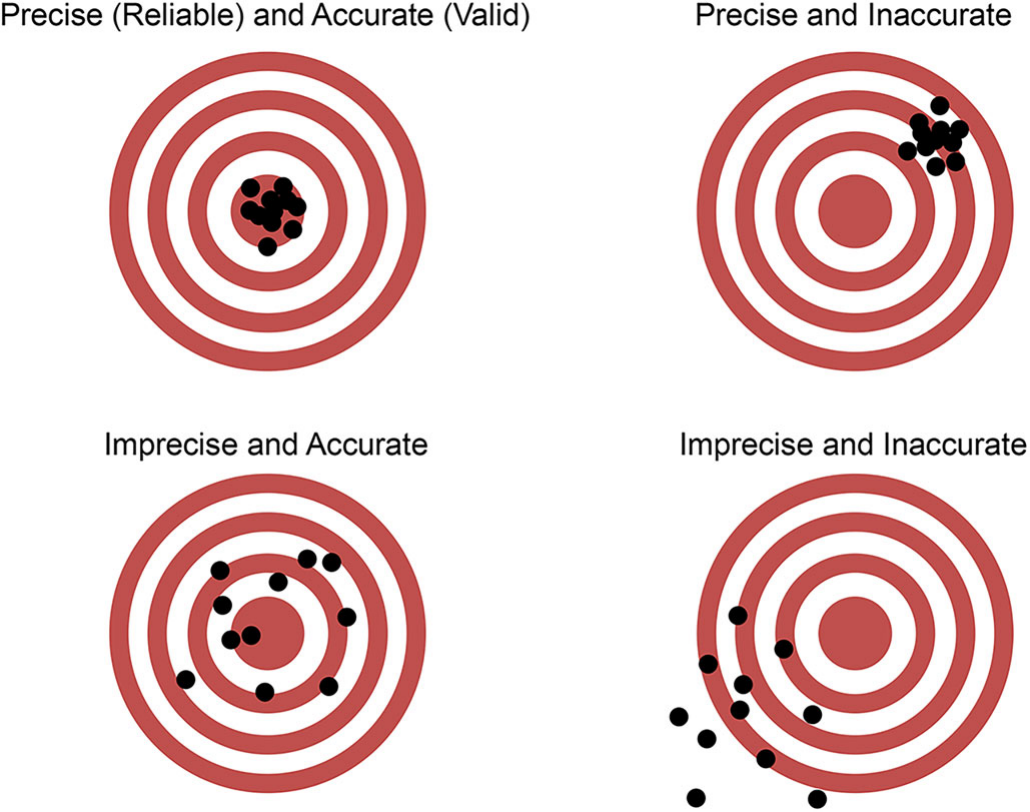
Precision (reliability) and accuracy (validity).

So what is bias? First and foremost, bias is a kind of error, but it is best explained more fully in the context of two statistical terms concerning the relationship among repeated measurements—“precision” (or “reliability”) and “accuracy” (or “validity”)—by way of analogy; see Figure 3, adapted from Russ Poldrack's online statistics resource, “Statistical Thinking for the 21st Century” (Poldrack, 2019). Imagine a person throwing darts at a target, the goal being to get as many darts as possible into the very center, or “bullseye,” which, in our scientific world, is often referred to as the “ground truth.” Our dart thrower's overall pattern of hits can be described by their precision and their accuracy. Precision is a measure of how consistently they throw—that is, how tightly the darts cluster near each other on the board. If they are tightly clustered, we say the aim is “precise,” regardless of how close they are to the bullseye. If they are widely scattered, we say they are “imprecise” (or “unreliable”). Accuracy, on the other hand, is a measure of how close the cluster is to the bullseye. All four combinations of precision and accuracy are depicted in Figure 3.

Bias is a failure of accuracy. It refers to any flaw in our experiment—from design to data collection to analysis—that causes systematic deviation from the ground truth. We can make the most precise measurements using state-of-the-art equipment, but if any part of our experimental procedure is biased, we will get it wrong. For scientists, “getting it right” is the whole idea, so bias is a bad thing. Unfortunately for science, there are many, many sources of bias in the world; so many that a team of scientists formed a consortium to catalog and study them. A recent survey of their website revealed 65 different types of bias that adversely affect outcomes, only a subset of which is represented in their “Big Table o’ Bias” (Nunan and Heneghan, 2018).

**Figure 4.**
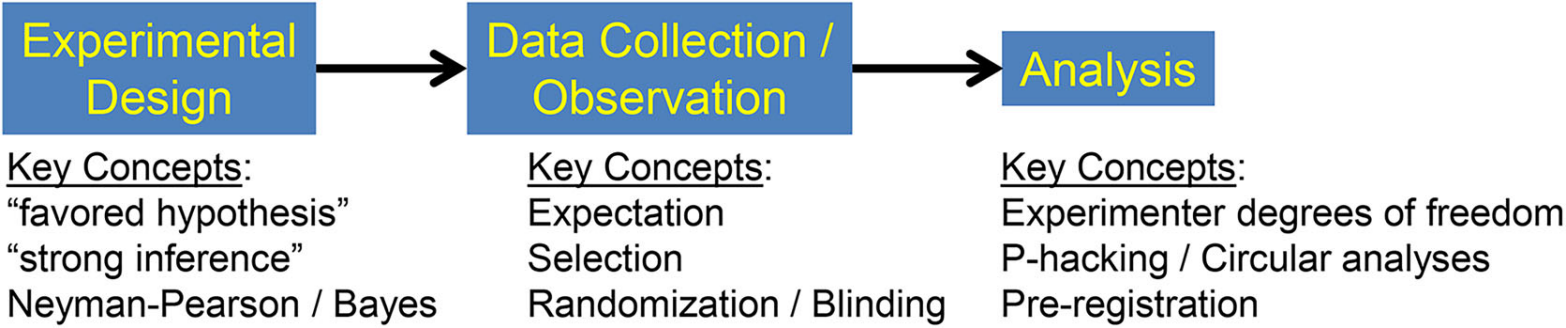
Stages of the scientific process at which confirmation bias can influence the outcome.

Confirmation bias can influence virtually all stages of a scientific project (Fig. 4): beginning with the initial phase of experimental design, it can blinker us to consider only our favored hypothesis, and extending to data analysis, where it motivates shoddy statistical practices like *p*-hacking (Simmons et al., 2011) and circular analysis (Kriegeskorte et al., 2009; Button, 2019). However, in this article, I will focus on the middle, where it can influence the observations we make during our experiments.

## Confirmation Bias

In 1960, the cognitive psychologist Peter Cathcart Wason conducted an experiment with 29 college students (Wason, 1960). He asked them to guess a general rule that generated instances of three numbers. He started them off with the sequence “2, 4, 6” as a positive example and then instructed them as follows: “Your aim is to discover this rule by writing down sets of three numbers, together with reasons for your choice of them. After you have written down each set, I shall tell you whether your numbers conform to the rule or not, and you can make a note of this outcome on the record sheet provided. There is no time limit but you should try to discover this rule by citing the minimum sets of numbers.”

To Wason's surprise, very few students discovered the rule—which was “any sequence of increasing numbers”—on their first attempt. Twenty-three of the 29 students got it wrong, even after proposing a fairly large number of instances. Most participants tended to fall into a rather narrow cognitive trap in which they formed a mental hypothesis such as, “even numbers ascending by 2” and then proceeded to put forth only examples that adhered to the hypothesis. But as you have probably figured out, in order to get to the more general rule (the “ground truth” here), they needed to propose some examples that violated the hypothesis. To describe this tendency for humans to generate, and seek out, information that confirms their hypotheses, Wason coined the term “confirmation bias.” It has been studied (and, yes, “confirmed”!) in a variety of different populations and settings ever since (Nickerson, 1998). The catalog of biases defines confirmation bias as, “The search for and use of information to support an individual's ideas, beliefs or hypotheses.”

In fact, the notion is a rather old one. In 1620, the English philosopher, Francis Bacon, sometimes referred to as “the father of empiricism,” described the tendency perfectly in his *Novum Organum* (Bacon, 1878):

“The human understanding when it has once adopted an opinion (either as being the received opinion or as being agreeable to itself) draws all things else to support and agree with it. And though there be a greater number and weight of instances to be found on the other side, yet these it either neglects and despises, or else by some distinction sets aside and rejects; in order that by this great and pernicious predetermination the authority of its former conclusions may remain inviolate.”

This, then, is confirmation bias—that “great and pernicious predetermination”—that is at the root of so much human error, both within science and beyond (Nickerson, 1998).

## Confirmation Bias Distorts Observations

Some of the first experiments to demonstrate this problem were carried out in the early 1960s by the psychologist, Robert Rosenthal, and his colleagues (Rosenthal and Fode, 1963; Rosenthal and Lawson, 1964). In one version, they asked students in their lab-based experimental psychology course to train rats on various tasks, such as using operant conditioning to get them to press a lever for a reward (Rosenthal and Lawson, 1964). The students were told that the purpose of the lab was to give them experience in working with experimental animals and to replicate some classic experimental findings. In addition, the students were divided into two groups: one group was told that they would be receiving rats that were “Skinner-box Bright,” meaning that they had been selected and bred to excel at the kinds of tasks the students would be training; the other group was told that their rats had been bred to do poorly at such tasks and were hence “Skinner-box Dull.” In reality, the two groups of rats were randomly selected from the same colony—the story about “bright” and “dull” rats was fabricated. Thus the belief state (“bright” vs “dull”) was the experimental manipulation; the students were the unwitting subjects.

**Figure 5.**
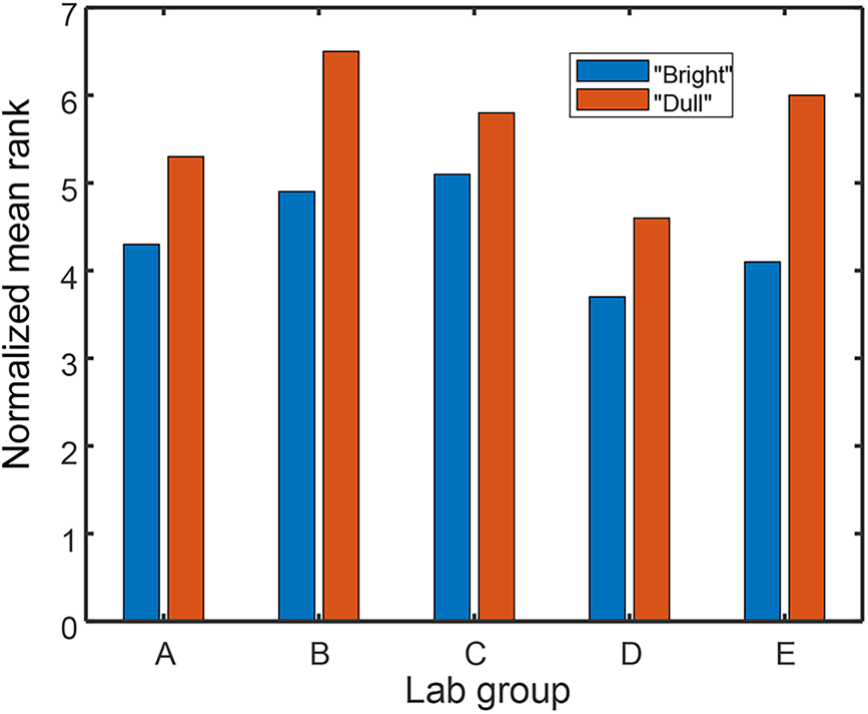
Data from Table 2 (p. 67) of Rosenthal and Lawson 1964. Source code for figure: https://github.com/rickborn/eNeuroCB2024/blob/main/eNeuro_figure_code/Python/figure5_Born_eNeuro.ipynb

The results were unambiguous: every group of students that believed they had “bright” rats obtained better performance from their animals than did the groups with “dull” rats (Fig. 5). Because the number of experiments performed by each laboratory group was relatively small, the results for individual pairs of lab groups were not statistically significant. However, because the investigators repeated the experiment with five different classes, they were able to show significant differences in the pooled data (*p* = 0.02). But this was just the beginning, and similar results have been obtained time and time and time again (Rosenthal and Rubin, 1978).

Note, however, that the above experiments were not just about observation—the differences in results produced by the subjects’ expectations could have been due to a number of additional factors, such as how they handled and trained the rats. But even when observation has been isolated, there remain large effects of confirmation bias. In one such experiment (Tuyttens et al., 2014), veterinary students were first trained to “score” social interactions between pigs, recognizing both positive interactions (e.g., play; nose-to-nose contact) and negative ones (e.g., head butting; ear or tail biting). They were then asked to score two videos of multiple pigs interacting in a pen. As with Rosenthal's early experiments, the students were told that one of the videos was of pigs that had been bred to be highly social (SBV+) and the other was of regular, “control” pigs. The trick was that both of the videos were essentially identical: one was the original video and the other was the same video but shown in mirror image with its brightness slightly modified and with made-up pen numbers and dates—modifications that altered none of the social content, but that would make it difficult for students to recognize that they were watching the same video. The beauty of the design was that each student served as their own control (i.e., all students watched both videos) and, because they had a lot of students (*n* = 157), they were able to counter-balance both the viewing order (i.e., half of the students viewed the “control” video 1st; the other half viewed it 2nd) and the video type (i.e., for half of the students, the original version was labeled as “SBV+”; for the other half it was labeled “control”), allowing them to control for these potential confounds.

Again, the results were clear. When study participants thought they were viewing the pro-social pigs, they reported more positive interactions and fewer negative ones, compared with when they believed they were viewing regular pigs [*p* < 0.0001, with a combined effect size, Cohen's *d*′, of 0.62; Tuyttens et al. (2014), their Fig. 2]. And similar biases have been seen for a variety of species and contexts: observations of panting in cattle (Tuyttens et al., 2014), qualitative assessments of the well-being of hens (Tuyttens et al., 2014), reports on classical conditioning in flatworms (Cordaro and Ison, 1963), and observations of nestmate recognition in ants (van Wilgenburg and Elgar, 2013).

One very interesting feature of the experiments described above is that none of the groups were in any way incentivized to obtain the expected result, yet they did anyway. Of course, many students are eager to please the professor, but they weren't, for example, told that they would get a better grade if they got a certain outcome. To me, this is scary, because in the actual science we practice in our labs, there is often a big incentive to get a certain result, whether because it supports our pet hypothesis or it increases the likelihood of a high-profile publication. Especially in translational studies, we often have a clearly desired outcome—who doesn't want to discover that their new experimental compound might cure a terrible disease? So, if anything, the psychological stakes in real lab experiments are much higher. To my knowledge, there are no systematic studies on whether the strength of the desire for a particular outcome influences the magnitude of the confirmation bias. But there is at least a hint of this in the aforementioned experiment with egg-laying hens. In this study, the investigators also surveyed the students as to their pre-existing opinions about the quality of life for chickens on organic versus conventional farms, and this measure correlated positively with the magnitude of the confirmation bias (Tuyttens et al., 2014). This seems like an important area for more systematic investigation.

## Mitigating Confirmation Bias through Randomization and Blinding

In general, the principles of good experimental design which help mitigate confirmation bias are:

1. Randomization: human choice is eliminated when assigning samples to treatment groups.
2. Blinding (or “masking”): those conducting the study, recording the data, and analyzing the data remain in the dark about which samples are in which treatment group.
3. Eliminating subjective assessments: using objective outcome measures that eliminate the opportunity for human bias.

Based on what we've learned about confirmation bias, you can probably already see the reasons for these safeguards, but I will expand upon them a bit here. Randomization removes the possibility of unconsciously selecting different participants for, say, a group destined to be treated with a drug of interest and the group destined to receive a placebo (controls). Almost everyone involved with a clinical trial of a new investigational drug, at some level, wants the drug to work—after all, we do what we do at least in part because we want to help people, and effective drugs do just that. So our inherent confirmation bias might, if we were the one making the assignments, cause us to unwittingly assign sicklier looking people to the placebo group and healthier looking people to the treatment group—a sure way to bias the study in favor of making the drug look like it works. Thus we remove this critical decision from human choice, and we make assignments using a random process, such as a computer algorithm or a coin toss. Once assignments are made, any knowledge of which patient belongs to which group might cause personnel involved in the study to treat labeled patients dissimilarly or to assess the outcome measures differently, thus introducing bias. For this reason, we use “blinding” (or “masking”) to keep investigators unaware of treatment group identity. In clinical trials that involve human patients, we use the term “double blind” to indicate that neither the patients nor the investigators know who got what.

## Randomization

Let’s imagine that we have 20 mice that we want to randomly assign to one of two groups: treatment with an experimental drug versus placebo (control). A simple and rigorous way to do this would be to simply flip a coin for each mouse in turn. If the coin comes up “heads,” we assign the mouse to the drug group; if “tails,” the placebo group. This is called “simple randomization.” You might be thinking, well, if the mice have been running around in their cage, they've already randomized themselves and I could just grab the first 10 and put them in one group and the rest in the other. While true in principle, there would be practical concerns with this method. Maybe the mice that are the easiest to catch are already less healthy (moving more slowly, perhaps) or are somehow less fearful of a hand reaching into the cage (Silberberg, 2024). For some experiments, this selection method would already introduce a bias. What if you were testing a drug for treating anxiety? By assigning the easiest-to-catch mice to the treatment group, you may already be biasing your result toward a false-positive or exaggerated drug effect. This is why it is important to preassign unique identification numbers to each subject and then perform the randomization based on these ID numbers.

**Figure 6.**
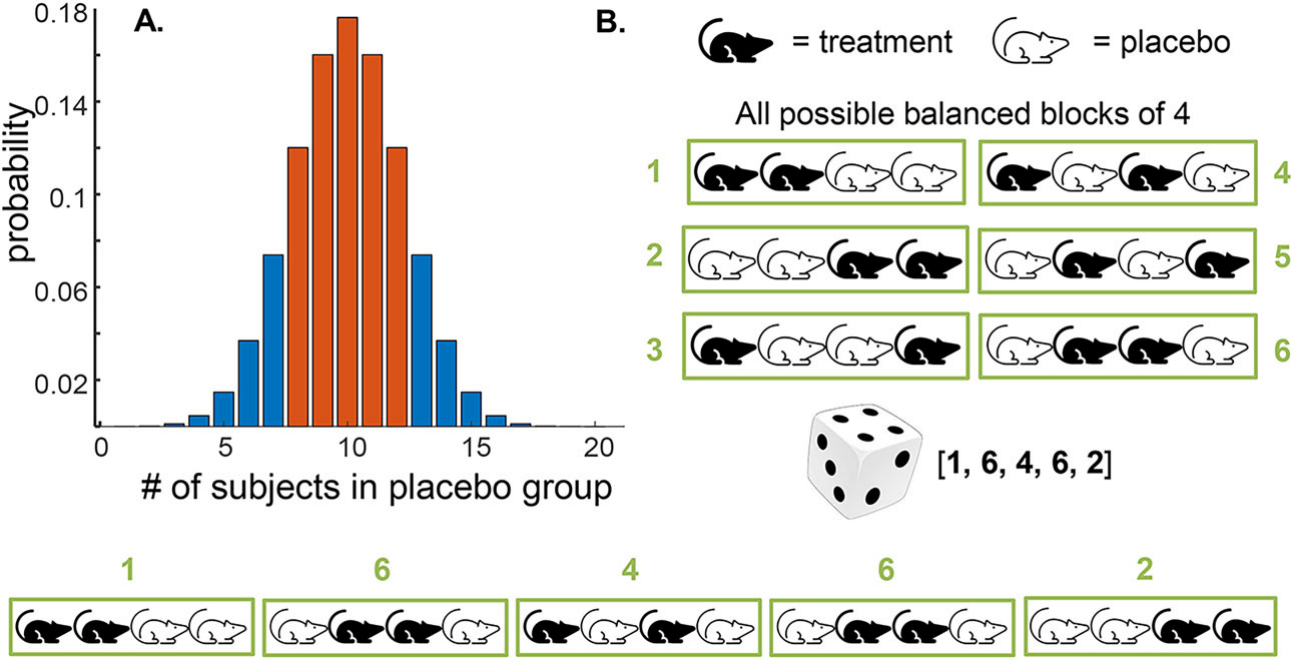
***A***, Binomial distribution, illustrating a problem of balancing group sizes with simple randomization. ***B***, Method for block randomization. Panel ***B*** adapted from Figure 2 of Kang et al., 2008. Source code for panel ***A***: https://github.com/rickborn/eNeuroCB2024/blob/main/eNeuro_figure_code/Python/figure6A_Born_eNeuro.ipynb

Simple randomization, as instantiated with the coin-toss method, has a drawback illustrated by the histogram in Figure 6*A*. Ideally, we'd like to have exactly 10 animals in each group, but the probability of this occurring with 20 coin tosses is <0.18. That is, over 80% of the times we used this method, we'd have unequal numbers of animals in the treatment and control groups. Even if we were willing to tolerate some unequalness—say a minimum of 8 animals in each group (red bars in Fig. 6*A*)—we'd still fail >25% of the time.

A way to defeat this problem is to randomize by blocks (Fig. 6*B*). To do this, we'd pick a reasonable block size (four is commonly used) and then prespecify all six possible blocks of four mice that each contain two treatment and two control animals. We would then use a random device that gives us an equal probability of choosing any one of the six blocks—a single die would be perfect. We'd roll the die 5 times (5 groups of 4 to assign 20 animals) and record the sequence of numbers (e.g., 1, 6, 4, 6, 2) and then we would string the corresponding blocks together to give us our assignment sequence, shown at the bottom of Figure 6*B*. This process guarantees that there will be 10 mice in each group while still maintaining reasonable randomness.

**Figure 7.**
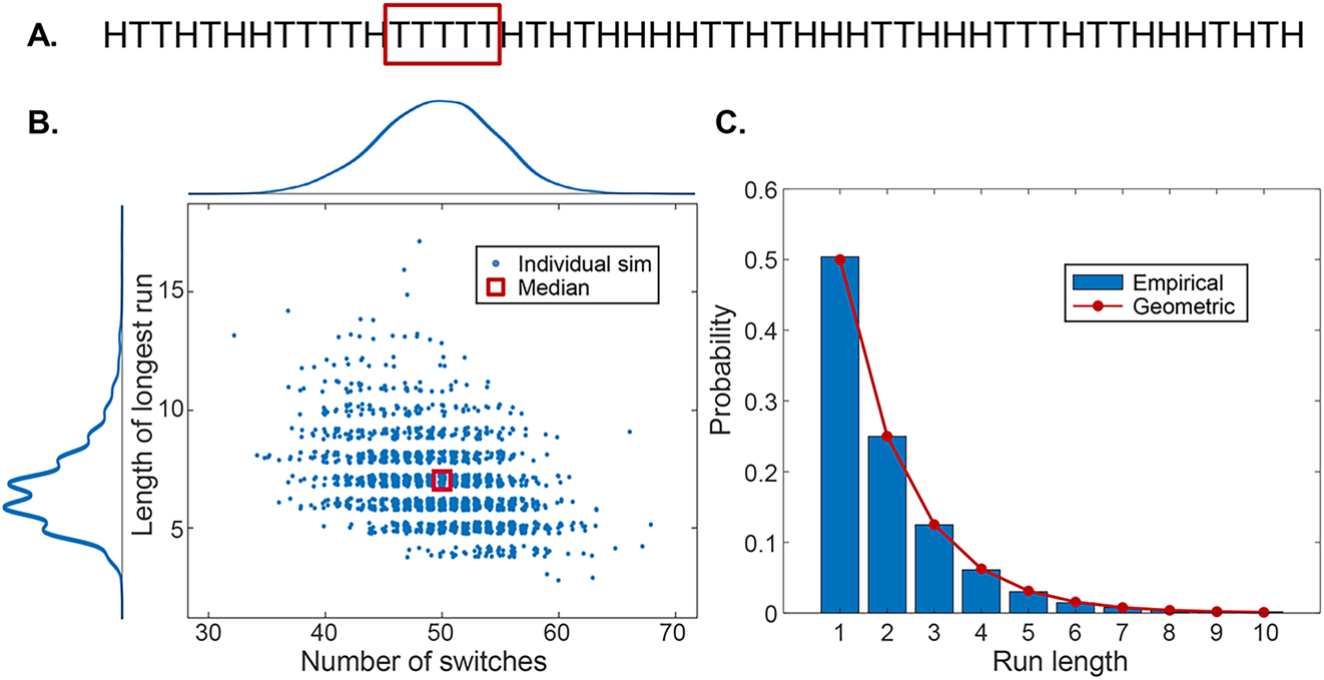
Binomial statistics. ***A***, One example of a simulation of tossing a fair coin 100 times. ***B***, Results from 2,000 such simulations. For each simulation, I recorded the number of “switches” (H followed by T or T followed by H) as well as the length of the longest run of consecutive tosses of the same value (H or T). ***C***, The probability of runs of different lengths is described by the geometric distribution. Source code for figure: https://github.com/rickborn/eNeuroCB2024/blob/main/eNeuro_figure_code/Python/figure7_Born_eNeuro.ipynb

The method of randomization by blocks also solves another drawback to simple randomization: long “runs” of a single assignment. Most of us have relatively poor intuition about the behavior of random processes (Tversky and Kahneman, 1971). More specifically, we tend to think that long runs of “heads” or “tails” should not occur very often, when, in reality, they do (Fig. 7). For large numbers of subjects assigned all at once, this might not be a problem. But imagine we are doing a clinical trial of a relatively rare disease, and we are enrolling participants as they appear in the clinic. Assigning seven or eight patients in a row to the same treatment group might not be ideal, particularly if there are slow temporal fluctuations in, say, the severity of the disease (perhaps due to changes in the ambient temperature or the presence of allergens). Block randomization limits this problem—in our previous example, the longest possible run would be 4, and this would occur only ∼5% of the time.

**Figure 8.**
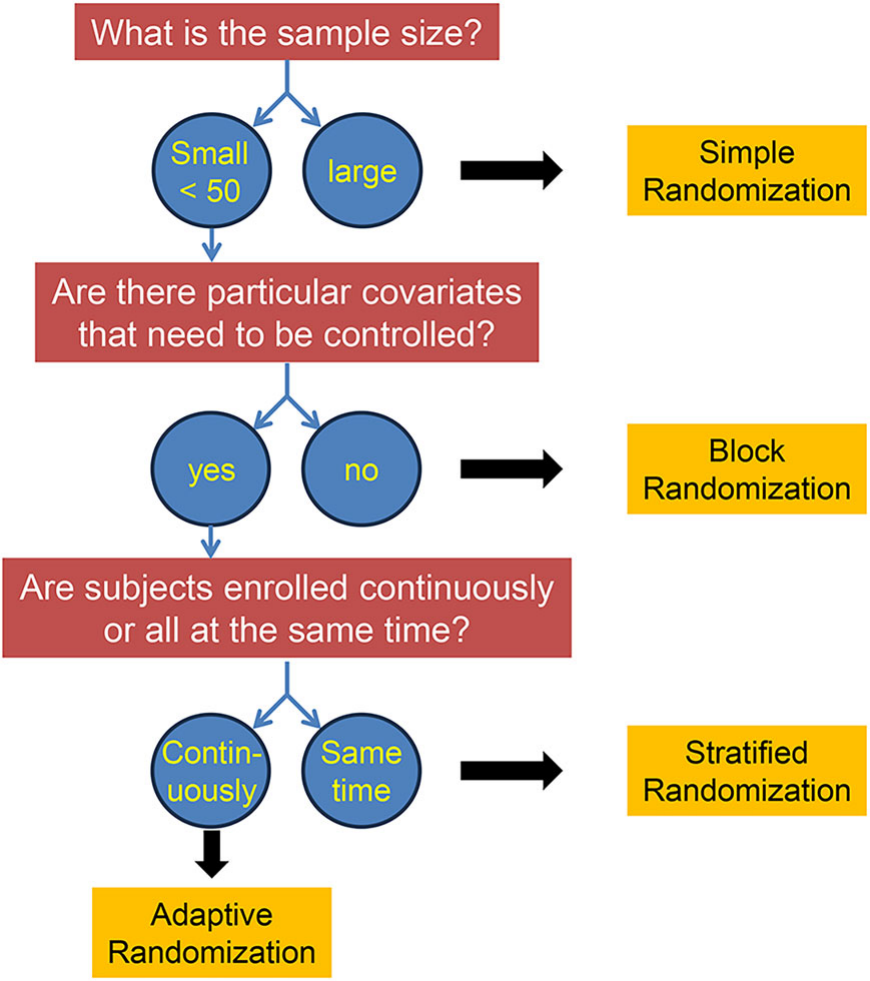
Flow chart to guide the choice of a method for randomization of subjects. Adapted from Figure 6 of Kang et al., 2008

For some studies, we might want to control particular covariates, such as the sex of the subjects, in which case we might preassign a group of subjects to their categories to make sure we have equal numbers in each, and then perform random assignment to treatment versus control within each category (i.e., M vs F). This is known as “stratified randomization.” It can only be used when we have all of our potential subjects identified in advance, so for continuously enrolled subjects there are fancier methods known as “adaptive randomization” that determine the assignment of each new subject as a function of both their personal characteristics (i.e., category membership for covariate balancing) and the assignments of previous subjects. To put these options in context, you can refer to the flow chart shown in Figure 8.

Note that there is some inevitable loss of randomness with methods other than simple randomization, so they must be used judiciously. Fortunately, they typically come into play for large randomized controlled trials (RCTs), where there is plenty of statistical expertise available. The details of these methods are beyond the scope of this tutorial, but the interested student can consult the following references (Kang et al., 2008; Alferes, 2012; Lim and In, 2019).

## Blinding

In an excellent overview of all aspects of blinding, particularly as it relates to clinical trials, Monaghan and colleagues define blinding (or “masking”) as “the process by which information that has the potential to influence study results is withheld from one or more parties involved in a research study” (Monaghan et al., 2021).

As we will see, blinding is simple in principle, but it is often difficult to do properly in practice. It requires very careful thinking about who needs to be blinded, when they need to be blinded, and, especially, about how to carry out the blinding so that it remains effective throughout the experiment. Unlike randomization, there is no flow chart or simple formula—you, as the experimentalist, must think very carefully about each phase of your experiment and apply the principle, keeping in mind that confirmation bias (CB) affects everyone.

The first question we need to ask is “Blind to what?” At a minimum, we want the people conducting the experiments (i.e., administering drug or placebo) and assessing the outcome (i.e., making observations and recording data) to be unaware of (1) to which group a given sample belongs and (2) which treatment a given sample received. Points 1 and 2 may sound like the same thing, but there is a subtle and important difference. If the person recording the data is aware that a given set of samples all belong to the same group—even if they don’t know which group—they may be biased in assigning outcome values that are more similar to each other. After all, they would know that all samples received the same treatment, whatever it was. But there is a potential third level of blinding as well—call it “max blinding”—which is when the outcome assessor has no idea about the aims or goals of the experiment. This is often hard to achieve, but a little thought shows why it could be valuable. In certain experiments, if there is a particularly strong effect of the treatment, there may be rather obvious differences between the treatment and control groups—differences directly related to the treatment and the outcome of interest, so there is no way for someone who knows the details of the experiment to remain effectively blind. On the one hand, this is a good thing, because it means the treatment effects aren't subtle and are therefore likely to be real. On the other hand, the failure of blinding, combined with the assessor's CB, could well lead to an exaggeration of the treatment effect size. A person who is “max blind” might still notice obvious behavioral differences, but since they would not know the goals of the experiment, they would be less likely to bias their observations one way or another.

Who needs to be blinded? In short, anyone who has a possible influence on the outcome of the experiment should be blinded to treatment groups. This includes the following: (1) participants (for human studies), (2) animal care staff (for animal studies), (3) experimentalists/clinicians, (4) data collectors, and (5) data analysts. When do personnel need to blinded? Simply put, during any part of the study where they could possibly influence the outcome.

How do you actually do the blinding? This will necessarily be different for different kinds of experiments. But for illustrative purposes, let's return to our example of assigning 20 mice to receive either drug or placebo. A typical way to carry out the blinding (along with the randomization) would be as follows.

First, you'd ask a colleague—someone you trust, but who is not involved in the experiment—to generate a random allocation sequence according to the method you specify, such as the block-random sequence shown in Figure 6*B*. They would then create a list of the identification numbers (IDs) of all of the animals to be used in your experiment, shuffle these numbers (either by using a standard permutation algorithm or by writing one animal ID# on each of 20 index cards and physically shuffling them, like a deck of cards) and then assign the shuffled animal ID#'s in order to the allocation sequence, keeping track of each animal's sequence number, ID#, and treatment group (Table 1). At the same time, the colleague should flip a coin and create a random “Group ID#” (e.g., If the coin comes up “heads,” they would assign the value “1” to every animal in the treatment group and “2” to those in the control group; vice versa for “tails.”). This last step is essential for anyone analyzing the data, for they must know how to group the data for statistical comparisons, but they shouldn't know the group identity (to prevent bias in analytical decisions, such as when to exclude data for technical reasons).

**Table 1.** A sample allocation table for 20 mice based on the method shown in Figure 6*B*

Allocation sequence #	Animal ID#	Group ID#	Group identity	Treatment administered
1	BL12SC7GM9	1	Rx	Drug X
2	BL12SC3GM8	1	Rx	Drug X
3	BL12SC8GM4	2	Ctrl	Normal saline
4	BL12SC5GM8	2	Ctrl	Normal saline
5	BL12SC7GM5	2	Ctrl	Normal saline
. . .				
20	BL12SC7GM6	1	Rx	Drug X

When it comes time to administer the treatment to the mice, you, the experimenter, would load up two groups of 10 syringes each containing either Drug X or saline, keeping them grouped in separate, labeled beakers, and then have your colleague label each syringe with the appropriate animal ID# and put them all together in one, unlabeled beaker. Your colleague would keep the allocation table in a safe place, hidden from you and anyone else involved in the experiment. All data collected would be referenced to the animal ID#; when it came time to analyze the data, the colleague would provide the analyst with a list of the group ID#'s corresponding to each animal ID# (but NOT the group identity). Only after the analyses were completed would they reveal to you which group received Drug X and which group received saline. These steps are summarized in Table 2.

**Table 2.** Steps in the example process of randomization and blinding

	What to do	Who does it	Comments
1	Generate a random allocation sequence	Colleague	Figure 6*B*
2	Shuffle all animal ID#s	Colleague	
3	Assign shuffled ID#s to allocation table	Colleague	Table 1
4	Generate group ID#s	Colleague	For analysis, e.g., Rx = 1, Ctrl = 2
5	Prepare syringes of substances to be administered	Experimenter	Don't label individual syringes! Group in separate beakers (X/Ctrl)
6	Label each syringe with an animal ID#	Colleague	Refer to allocation table
7	Collect data, referenced to animal ID#	Experimenter	
8	Provide analyst with group ID#s	Colleague	Statistical comparison of groups
9	Reveal group identity after analysis is complete	Colleague	

As previously mentioned, all of these principles are conceptually simple, but implementing them effectively requires common sense, attention to detail, and continued vigilance. It is impossible in this brief tutorial to consider all of the many situations that pose challenges to effective blinding. For further details, the reader can consult excellent reviews on blinding for both human (Monaghan et al., 2021) and animal (Karp et al., 2022) studies. Even in situations where blinding is virtually impossible, as in RCTs involving psychedelic drugs, the influence of confirmation bias can be mitigated by careful experimental design and by including measures of expectancy and unblinding (Muthukumaraswamy et al., 2021).

## Does Blinding Make a Difference?

We've already seen powerful evidence from psychology experiments that any time there is any sort of hope, suggestion, or expectation of a particular outcome—even when observers don't necessarily have a big stake in that outcome—humans are highly biased. But these have all been experiments on nonscientists in highly controlled settings. Perhaps scientists are less susceptible to CB. Maybe it's not such a big deal for real experiments.

But there is ample data indicating that blinding matters “in real life.” For example, the results of one meta-analysis show that failing to randomize allocation or use blinding inflated effect sizes of neurobehavioral scores in animal models of multiple sclerosis (Vesterinen et al., 2010). Failure to randomize allocation to group produced a doubling of the average reported effect size (*p* < 0.002), and a lack of blinding for the outcome assessment increased the average effect size by ∼40% (*p* < 0.002). Of course, since the ground truth isn't known, all the authors can show is a difference. In principle, it could be that blinding artificially decreases effect sizes. But given what we know about confirmation bias, is this a reasonable interpretation? Moreover, it fails to account for why multiple studies have not only found differences in effect sizes between studies that blind and those that don't, they are always in the same direction, favoring larger treatment effects in unblinded studies.

In another meta-analysis of many experiments testing a single experimental drug, NXY-059, for treating experimental stroke (Macleod et al., 2008), the drug was reported to be 30% more effective in studies that were not randomized or blinded than in studies that were. Another meta-analysis of 290 animal research studies, presented at an academic emergency medicine meeting (Bebarta et al., 2003), found that studies that used neither randomization nor blinding were over five times more likely to obtain a positive outcome than studies that used both design features. Comparable results have been reported from other analyses examining preclinical studies in animal models (Rooke et al., 2011; Hirst et al., 2014; Sena et al., 2014).

A similar picture emerges from randomized clinical trials (RCTs) in humans. In one especially valuable series of studies, Hróbjartsson and colleagues performed meta-analyses on RCTs that were specifically selected for having blinded and unblinded assessors within the same study, thus controlling for the many possible confounds involved in between-studies comparisons. Regardless of the type of outcome measure used, they found that unblinded assessment of the outcome produced larger effect sizes favoring the treatment: by 68% for measurement scale outcomes (Hróbjartsson et al., 2013), by 36% in studies using binary outcomes (Hróbjartsson et al., 2012), and by 27% in studies with time-to-event outcomes (Hróbjartsson, Thomsen, et al., 2014). They found a similar problem with participant-reported outcomes when the patients were not blinded, with a moderate bias of 0.56 standard deviations in favor of the treatment (Hróbjartsson, Emanuelsson, et al., 2014). Similar results have been reported by other investigative teams (Moher et al., 1998; Jüni et al., 2001; Bero et al., 2007; Savović et al., 2012).

In summary, if we care about “getting it right,” we ought to be blinding.

## How Good Is Your Mask?

You've done all the blinding properly, and now it comes time for you to evaluate the effect of Drug X. What if there is something subtly “different” about the two groups—something that is completely unrelated to the measure of interest, but that, nonetheless, gives away the group identity of the animal? This is what professional poker players call a “tell”—anything that gives away a player's emotional response to the cards they've been dealt. Is it possible that your mutant has a tell as well? How would you figure this out?

It's not difficult to come up with a long list of things that could potentially unblind an experiment. Again, there is no simple cookbook solution to the problem, so the experimentalist must sit down and carefully consider the possibilities that are most relevant to their particular experiment. And then they must try to come up with ways to defeat them.

The idea of assessing the efficacy of blinding is, like many things related to blinding, conceptually rather simple. For example, in the case of an RCT, one could just ask the patients whether they thought they got the active treatment or not (there are a number of possible variations on how to do this; reviewed in Muthukumaraswamy et al., 2021; pp. 1139–40). But is this wise? Simply asking the question encourages the patients to think more deeply about which treatment group they're in, which runs the risk of introducing more bias. Of course, one might ask the question near the end of the trial, after all of the data had been collected. But, in fact, the most recent guidelines on how to conduct clinical trials, the CONSORT statement (Schulz et al., 2010), do not recommend that investigators explicitly assess the effectiveness of blinding in study participants. And similar issues arise in laboratory experiments: by asking the data collector to attempt to identify each group, we might actually exacerbate unblinding and thereby increase bias. I don't think there is an obvious recommendation to be made on this score, but I do think that insofar as we become aware of unblinding in our experiments, we are obligated to report it in our papers and discuss the potential bias that might have been (even unwittingly) introduced. This is a part of the “utter honesty” that Richard Feynman recommended in his famous “Cargo Cult Science” commencement address at CalTech (Feynman, 1974):

“It's a kind of scientific integrity, a principle of scientific thought that corresponds to a kind of utter honesty—a kind of leaning over backwards. For example, if you're doing an experiment, you should report everything that you think might make it invalid—not only what you think is right about it: other causes that could possibly explain your results; and things you thought of that you've eliminated by some other experiment, and how they worked—to make sure the other fellow can tell they have been eliminated.”

## Are Anti-CB Design Features Widely Used?

In RCTs that involve human subjects, these features are *de rigueur*, enforced by panels of experts, including statisticians, that monitor such trials. But when it comes to preclinical studies in animal models, the answer is a resounding, “No.” The data in Table 3, adapted from (Sena et al., 2014), tells us about all we need to know. They surveyed over 2,000 published papers in which experimental therapies were tested in animal models of various neurological diseases. Although there was some variability across different disease models, overall, only 21% of the studies reported the use of blinding during outcome assessment, and even fewer (16%) used randomization when allocating animals to the treatment versus control groups. Note that this meta-analysis was able to assess only whether or not studies reported using various bias reduction measures. It is conceivable that some of the studies used randomization and/or blinding but failed to report it in the Methods section. However, given the extra effort required to implement these antibias measures, and the general awareness that they improve the quality of a study, it seems unlikely that under-reporting is a major factor.

**Table 3.** Frequency of blinding and randomization in various studies of animal disease models

Disease modeled	Number of papers	Blind outcome assessment, # % [95% CI]		Randomized group allocation, # % [95% CI]	
Alzheimer's disease	428	95	22 [18, 26]	67	16 [12, 19]
Multiple sclerosis	1,117	178	16 [14, 18]	106	9 [8, 11]
Parkinson's disease	252	38	15 [11, 20]	40	16 [12, 21]
Intracerebral bleed	88	43	49 [38, 60]	27	31 [21, 41]
Focal ischemia	260	87	33 [28, 40]	100	38 [32, 45]
Totals	2,145	441	**21 [19, 22]**	340	**16 [14, 17]**

Data from Table 1 of Sena et al., 2014.

## Tools for Removing Subjectivity

Blinding is essential in studies for which a subjective assessment of behavior or other outcome is used. In the field of neuroscience, however, there are many experiments where subjectivity can be completely removed from, at least, the preanalytical phases of the experiment. For example, in my own fields of systems neuroscience and visual neurophysiology, virtually all phases of the experiment, up to and including data collection, are controlled by computers. The experimenter merely specifies the parameters of the experiment—the range of visual stimuli to be presented, the behavioral contingencies, causal interventions (e.g., microstimulation or optogenetic activation), etc.—and then the code used to run the experiment generates a table of all possible trial types and uses an internal random number generator to select the order of the individual trials. In addition, the computer automatically records the data, including neuronal signals (e.g., action potentials), images of brain activity (e.g., 2-photon Ca^++^ signals), operant responses (e.g., lever presses, water-spout licks), and behavioral output (e.g., eye movements, facial expressions).

Appropriately coded, computer-controlled experiments offer good protection against confirmation bias during the treatment and data acquisition phases of an experiment. Often, however, the Achilles heel with respect to CB is the analysis phase, where the resulting high-dimensional data sets offer many opportunities for subjective decisions—what Simmons et al., (2011) refer to as “researcher degrees of freedom”—including when to exclude data, whether to subset the data, the choice of analytical models, and many others. As the economist Ronald Coase famously said, “If you torture the data long enough, it will confess.” (Good, 1972), and this sort of “*p*-hacking” (Simonsohn et al., 2014) is best combatted by not conflating exploratory (i.e., “torturing the data”) and confirmatory (i.e., hypothesis testing) analyses. That is, when performing a hypothesis test, one should state the specific hypothesis “up front” (using pre-registration, where appropriate) and then conduct the final analysis in a blind fashion.

## Objective Assessment of Behavior

Many experiments in neuroscience require a behavioral assessment of the outcome. Because these types of evaluation are highly subjective, as we have already seen, they are prone to confirmation bias on the part of the assessor. Moreover, because behavior has so many dimensions, there may be subtle differences between treatment groups (“tells”—see above) that are unrelated to the outcome of interest but that may nonetheless render blinding ineffective. Fortunately, thanks to improved sensor technology, the availability of inexpensive high-speed video cameras and advances in machine learning, very sophisticated, quantitative assessments of behavior that largely eliminate human judgement are increasingly possible. One such tool that is widely used, “DeepLabCut” (Mathis et al., 2018), exploits a deep convolutional network that has been pretrained to perform object recognition. This allows the user to rapidly train it to recognize and track specific body parts, such as a the joints on a mouse's paw, by manually labeling only a few hundred video frames. Because the only subjective step—the manual labeling—can be done without experimental context and blind to the group identity of any particular animal, the network's output is not biased toward a particular outcome. Other similar methods exist (Wiltschko et al., 2015; Pereira et al., 2022) and new ones are constantly being developed. The details of these methods are beyond the scope of this tutorial, but the student should be aware that they exist and make every possible effort to use them in their research when appropriate. While machine learning algorithms are not immune to bias, they can eliminate the confirmation bias that concerns us here.

## Say What You Did!

After you've gone to all the trouble to incorporate antibias features in your experiment, it's critical to let the world know that you did so. This means that you need to accurately report them in your Methods section. Fortunately, there are two valuable resources that provide detailed instructions on how to do this properly. For preclinical studies, there are the ARRIVE guidelines, now in their 2nd version (Kilkenny et al., 2010; Percie du Sert et al., 2020), and their clinical-trial predecessor, the CONSORT guidelines (Schulz et al., 2010). For example, ARRIVE (which is an acronym for Animals in Research: Reporting In Vivo Experiments) lists its “essential 10”: ten items that are “the basic minimum that must be included in any manuscript describing animal research.” “Randomization” is item #4 and “Blinding/Masking” is item #5. Each item consists of a concise instruction, followed by a detailed explanation, including justification and references to the relevant literature, along with specific examples of proper reporting practice. The page on blinding/masking, for example, instructs authors to “Describe who was aware of the group allocation at the different stages of the experiment (during the allocation, the conduct of the experiment, the outcome assessment, and the data analysis).” Importantly, the accompanying page provides real-world examples of appropriate reporting for situations in which blinding both was and was not possible (Hsieh et al., 2017):

“Investigators could not be blinded to the mouse strain due to the difference in coat colors, but the three-chamber sociability test was performed with ANY-maze video tracking software (Stoelting, Wood Dale, IL, USA) using an overhead video camera system to automate behavioral testing and provide unbiased data analyses. The one-chamber social interaction test requires manual scoring and was analyzed by an individual with no knowledge of the questions.”

**Figure 9.**
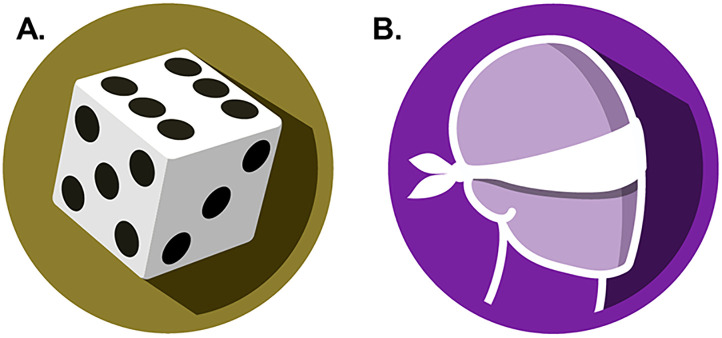
Rigor icons for (***A***) randomization and (***B***) blinding. PNG files freely available from: https://www.ninds.nih.gov/current-research/*trans*-agency-activities/rigor-transparency/rigor-champions-and-opportunities/maximizing-data-transparency-rigor-icons

As a visual companion to the reporting guidelines, a group of scientists affiliated with the National Institute of Neurological Disorders and Stroke has created downloadable “rigor icons” (Silberberg et al., 2017) that can be added to figures to convey critical details of experimental design at a glance. The icons represent a “core set of reporting standards,” including randomization and blinding (Fig. 9).

## Summary and Conclusions

My hope is that this brief tutorial has gone some way toward convincing you that confirmation bias is a serious problem. The bottom line is that we are surprisingly good at fooling ourselves, especially when we have an incentive to do so. Fortunately, there are several features of good experimental design that eliminate the need for self-trust, especially randomization and blinding, but they require thought, effort, and careful implementation to be effective.

In closing, I'd like to share my favorite quote on the phenomenon of confirmation bias by the greatest biologist of all time. Here's what Charles Darwin had to say in his autobiography (Darwin 2005):

“I had, also, during many years followed a golden rule, namely, that whenever a published fact, a new observation or thought came across me, which was opposed to my general results, to make a memorandum of it without fail and at once; for I had found by experience that such facts and thoughts were far more apt to escape from the memory than favourable ones.”

Darwin didn't want to fool himself, and neither should you.

## Data Availability

The code used to create Figures 5, 6*A* and 7 can be found at https://github.com/rickborn/eNeuroCB2024. The repository contains versions in both MATLAB and Python.

## Synthesis

Reviewing Editor: Arvind Kumar, KTH Royal Institute of Technology Decisions are customarily a result of the Reviewing Editor and the peer reviewers coming together and discussing their recommendations until a consensus is reached. When revisions are invited, a fact-based synthesis statement explaining their decision and outlining what is needed to prepare a revision will be listed below. The following reviewer(s) agreed to reveal their identity: NONE.

The article addresses an important problem associated with hypotheses-driven research. The authors has provided an excellent tutorial on this topic and how one may avoid the bias.

While the paper is already in publishable state, we thought it maybe worth adding some discussion about computerized experiments where almost all control is in the hands of a computer.

## References

[B1] Alferes VR (2012) *Methods of randomization in experimental design*. Thousand Oaks, CA: SAGE Publications, Inc.

[B2] Bacon F (1878) *Novum organum*. Oxford: Clarendon press.

[B3] Bebarta V, Luyten D, Heard K (2003) Emergency medicine animal research: does use of randomization and blinding affect the results? Acad Emerg Med 10:684–687. 10.1111/j.1553-2712.2003.tb00056.x 12782533

[B4] Bero L, Oostvogel F, Bacchetti P, Lee K (2007) Factors associated with findings of published trials of drug-drug comparisons: why some statins appear more efficacious than others. PLoS Med 4:e184. 10.1371/journal.pmed.0040184 17550302 PMC1885451

[B5] Button KS (2019) Double-dipping revisited. Nat Neurosci 22:688–690. 10.1038/s41593-019-0398-z 31011228

[B6] Cordaro L, Ison JR (1963) Psychology of the scientist: X. Observer bias in classical conditioning of the planarian. Psychol Rep 13:787–789. 10.2466/pr0.1963.13.3.787

[B7] Darwin F (2005) *The autobiography of Charles Darwin: from the life and letters of Charles Darwin*, Ed. 1. Fairfield, IA: 1st World Library.

[B8] Feynman R (1974) *Cargo cult science* [Commencement address]. https://calteches.library.caltech.edu/51/2/CargoCult.htm

[B9] Good IJ (1972) Statistics and today’s problems. Am Stat 26:11–19. 10.2307/2682859

[B10] Gregory RL (1997) *Eye and brain: the psychology of seeing*, Ed. 5. Princeton, NJ: Princeton University Press.

[B11] Hirst JA, Howick J, Aronson JK, Roberts N, Perera R, Koshiaris C, Heneghan C (2014) The need for randomization in animal trials: an overview of systematic reviews. PLoS One 9:e98856. 10.1371/journal.pone.0098856 24906117 PMC4048216

[B12] Hróbjartsson A, Emanuelsson F, Skou Thomsen AS, Hilden J, Brorson S (2014) Bias due to lack of patient blinding in clinical trials. A systematic review of trials randomizing patients to blind and nonblind sub-studies. Int J Epidemiol 43:1272–1283. 10.1093/ije/dyu115 24881045 PMC4258786

[B13] Hróbjartsson A, Thomsen ASS, Emanuelsson F, Tendal B, Hilden J, Boutron I, Ravaud P, Brorson S (2012) Observer bias in randomised clinical trials with binary outcomes: systematic review of trials with both blinded and non-blinded outcome assessors. BMJ 344:e1119. 10.1136/bmj.e1119 22371859

[B14] Hróbjartsson A, Thomsen ASS, Emanuelsson F, Tendal B, Hilden J, Boutron I, Ravaud P, Brorson S (2013) Observer bias in randomized clinical trials with measurement scale outcomes: a systematic review of trials with both blinded and nonblinded assessors. CMAJ 185:E201–E211. 10.1503/cmaj.120744 23359047 PMC3589328

[B15] Hróbjartsson A, Thomsen ASS, Emanuelsson F, Tendal B, Rasmussen JV, Hilden J, Boutron I, Ravaud P, Brorson S (2014) Observer bias in randomized clinical trials with time-to-event outcomes: systematic review of trials with both blinded and non-blinded outcome assessors. Int J Epidemiol 43:937–948. 10.1093/ije/dyt270 24448109

[B16] Hsieh LS, Wen JH, Miyares L, Lombroso PJ, Bordey A (2017) Outbred CD1 mice are as suitable as inbred C57BL/6J mice in performing social tasks. Neurosci Lett 637:142–147. 10.1016/j.neulet.2016.11.035 27871995 PMC5203811

[B17] Jüni P, Altman DG, Egger M (2001) Systematic reviews in health care: assessing the quality of controlled clinical trials. BMJ 323:42–46. 10.1136/bmj.323.7303.42 11440947 PMC1120670

[B18] Kahneman D, Tversky A (1996) On the reality of cognitive illusions. Psychol Rev 103:582–591; discusion 592-596. 10.1037/0033-295x.103.3.582 8759048

[B19] Kang M, Ragan BG, Park J-H (2008) Issues in outcomes research: an overview of randomization techniques for clinical trials. J Athl Train 43:215–221. 10.4085/1062-6050-43.2.215 18345348 PMC2267325

[B20] Karp NA, Pearl EJ, Stringer EJ, Barkus C, Ulrichsen JC, du Sert NP (2022) A qualitative study of the barriers to using blinding in in vivo experiments and suggestions for improvement. PLoS Biol 20:e3001873. 10.1371/journal.pbio.3001873 36395326 PMC9714947

[B21] Kilkenny C, Browne WJ, Cuthill IC, Emerson M, Altman DG (2010) Improving bioscience research reporting: the ARRIVE guidelines for reporting animal research. PLoS Biol 8:e1000412. 10.1371/journal.pbio.1000412 20613859 PMC2893951

[B22] Kriegeskorte N, Simmons WK, Bellgowan PSF, Baker CI (2009) Circular analysis in systems neuroscience: the dangers of double dipping. Nat Neurosci 12:535–540. 10.1038/nn.2303 19396166 PMC2841687

[B23] Lim C-Y, In J (2019) Randomization in clinical studies. Korean J Anesthesiol 72:221–232. 10.4097/kja.19049 30929415 PMC6547231

[B24] Macleod MR, van der Worp HB, Sena ES, Howells DW, Dirnagl U, Donnan GA (2008) Evidence for the efficacy of NXY-059 in experimental focal cerebral ischaemia is confounded by study quality. Stroke 39:2824–2829. 10.1161/STROKEAHA.108.515957 18635842

[B25] Mathis A, Mamidanna P, Cury KM, Abe T, Murthy VN, Mathis MW, Bethge M (2018) Deeplabcut: markerless pose estimation of user-defined body parts with deep learning. Nat Neurosci 21:1281–1289. 10.1038/s41593-018-0209-y 30127430

[B26] Moher D, Pham B, Jones A, Cook DJ, Jadad AR, Moher M, Tugwell P, Klassen TP (1998) Does quality of reports of randomised trials affect estimates of intervention efficacy reported in meta-analyses? Lancet 352:609–613. 10.1016/S0140-6736(98)01085-X 9746022

[B27] Monaghan TF, Agudelo CW, Rahman SN, Wein AJ, Lazar JM, Everaert K, Dmochowski RR (2021) Blinding in clinical trials: seeing the big picture. Medicina (Kaunas) 57:647. 10.3390/medicina57070647 34202486 PMC8308085

[B28] Muthukumaraswamy SD, Forsyth A, Lumley T (2021) Blinding and expectancy confounds in psychedelic randomized controlled trials. Expert Rev Clin Pharmacol 14:1133–1152. 10.1080/17512433.2021.1933434 34038314

[B29] Nickerson RS (1998) Confirmation bias: a ubiquitous phenomenon in many guises. Rev Gen Psychol 2:175–220. 10.1037/1089-2680.2.2.175

[B30] Nunan D, Heneghan C (2018) *Catalogue of Bias*. https://catalogofbias.org/about/

[B31] Percie du Sert N, et al. (2020) The ARRIVE guidelines 2.0: updated guidelines for reporting animal research. PLoS Biol 18:e3000410. 10.1371/journal.pbio.3000410 32663219 PMC7360023

[B32] Pereira TD, et al. (2022) SLEAP: a deep learning system for multi-animal pose tracking. Nat Methods 19:486–495. 10.1038/s41592-022-01426-1 35379947 PMC9007740

[B33] Poldrack RA (2019) *Statistical thinking for the 21st century*. Stanford, CA: Open Source Book. https://github.com/statsthinking21/statsthinking21-core

[B34] Rooke EDM, Vesterinen HM, Sena ES, Egan KJ, Macleod MR (2011) Dopamine agonists in animal models of Parkinson’s disease: a systematic review and meta-analysis. Parkinsonism Relat Disord 17:313–320. 10.1016/j.parkreldis.2011.02.010 21376651

[B35] Rosenthal R, Fode K (1963) The effect of experimenter bias on the performance of the albino rat. Behav Sci 8:183–189.

[B36] Rosenthal R, Lawson R (1964) A longitudinal study of the effects of experimenter bias on the operant learning of laboratory rats. J Psychiatr Res 2:61–72. 10.1016/0022-3956(64)90003-2 14177091

[B37] Rosenthal R, Rubin D (1978) Interpersonal expectancy effects: the first 345 studies. Behav Brain Sci 1:377–386. 10.1017/S0140525X00075506

[B38] Savović J, et al. (2012) Influence of reported study design characteristics on intervention effect estimates from randomized, controlled trials. Ann Intern Med 157:429–438. 10.7326/0003-4819-157-6-201209180-00537 22945832

[B39] Schulz KF, Altman DG, Moher D, CONSORT Group. (2010) CONSORT 2010 statement: updated guidelines for reporting parallel group randomised trials. BMJ 340:c332. 10.1136/bmj.c332 20332509 PMC2844940

[B40] Sena ES, Currie GL, McCann SK, Macleod MR, Howells DW (2014) Systematic reviews and meta-analysis of preclinical studies: why perform them and how to appraise them critically. J Cereb Blood Flow Metab 34:737–742. 10.1038/jcbfm.2014.28 24549183 PMC4013765

[B41] Silberberg SD (2024) *Attaining a bright future for biomedical research [Seminar]*. Boston, MA: Scientific Rigor Seminar Series, Kirby Neurobiology Center, Harvard Medical School.

[B42] Silberberg SD, Crawford DC, Finkelstein R, Koroshetz WJ, Blank RD, Freeze HH, Garrison HH, Seger YR (2017) Shake up conferences. Nature 548:153–154. 10.1038/548153a 28796229

[B43] Simmons JP, Nelson LD, Simonsohn U (2011) False-positive psychology: undisclosed flexibility in data collection and analysis allows presenting anything as significant. Psychol Sci 22:1359–1366. 10.1177/0956797611417632 22006061

[B44] Simonsohn U, Nelson LD, Simmons JP (2014) P-curve: a key to the file-drawer. J Exp Psychol Gen 143:534–547. 10.1037/a0033242 23855496

[B45] Tuyttens FAM, De Graaf S, Heerkens JLT, Jacobs L, Nalon E, Ott S, Stadig L, Van Laer E, Ampe B (2014) Observer bias in animal behaviour research: can we believe what we score, if we score what we believe? Anim Behav 90:273–280. 10.1016/j.anbehav.2014.02.007

[B46] Tversky A, Kahneman D (1971) Belief in the law of small numbers. Psychol bull 76:105–110. 10.1037/h0031322

[B47] van Wilgenburg E, Elgar MA (2013) Confirmation bias in studies of nestmate recognition: a cautionary note for research into the behaviour of animals. PLoS One 8:e53548. 10.1371/journal.pone.0053548 23372659 PMC3553103

[B48] Vesterinen HM, Sena ES, ffrench-Constant C, Williams A, Chandran S, Macleod MR (2010) Improving the translational hit of experimental treatments in multiple sclerosis. Mult Scler 16:1044–1055. 10.1177/1352458510379612 20685763

[B49] Wason PC (1960) On the failure to eliminate hypotheses in a conceptual task. Q J Exp Psychol 12:129–140.

[B50] Weiss Y, Simoncelli EP, Adelson EH (2002) Motion illusions as optimal percepts. Nat Neurosci 5:598–604. 10.1038/nn0602-858 12021763

[B51] Wiltschko AB, Johnson MJ, Iurilli G, Peterson RE, Katon JM, Pashkovski SL, Abraira VE, Adams RP, Datta SR (2015) Mapping sub-second structure in mouse behavior. Neuron 88:1121–1135. 10.1016/j.neuron.2015.11.031 26687221 PMC4708087

[B52] Wolfe U, Maloney LT, Tam M (2005) Distortions of perceived length in the frontoparallel plane: tests of perspective theories. Percept Psychophys 67:967–979. 10.3758/bf03193624 16396006

